# Health Risk Assessment of Heavy Metals in Rice to the Population in Zhejiang, China

**DOI:** 10.1371/journal.pone.0075007

**Published:** 2013-09-06

**Authors:** Zhu Huang, Xiao-Dong Pan, Ping-Gu Wu, Jian-Long Han, Qing Chen

**Affiliations:** 1 Department of Ophthalmology, the First Affiliated Hospital, College of Medicine, Zhejiang University, Hangzhou, China; 2 Zhejiang Provincial Center for Disease Control and Prevention, Hangzhou, China; National Research Council, Italy

## Abstract

Environmental pollution with toxic heavy metals can lead to the possible contamination of the rice. Selected metals (As, Cd, Hg and Pb) and their accumulation in rice collected from Zhejiang, China were analyzed to evaluate the potential health risk to the local adults and children. The mean levels found in rice were as follows: As, 0.080 mg/kg; Cd, 0.037 mg/kg; Hg, 0.005 mg/kg; Pb, 0.060 mg/kg. The estimated daily intakes (EDIs) were calculated in combination of the rice consumption data. The mean intakes of As, Cd, Hg and Pb through rice were estimated to be 0.49, 0.23, 0.03 and 0.37 µg/kg bw/day for adults, and 0.34, 0.29, 0.04 and 0.47 µg/kg bw/day for children. The 97.5th percentile (P97.5) daily intakes of As, Cd, Hg and Pb were 1.02, 0.64, 0.37 and 1.26 µg/kg bw/day for adults, and 0.63, 0.83, 0.47 and 1.63 µg/kg bw/day for children. The risk assessment in mean levels showed that health risk associated with these elements through consumption of rice was absent. However, estimates in P97.5 level of Cd and Pb for children, and Hg for adults have exceeded the respective safe limits.

## Introduction

Rice, *Oryza sativa*, is a major food in Asian countries, where its production constitutes over 90% of the global production [1]. China, the largest rice producer in the world, owns the output accounting for 30.7% of the whole [2]. In the Southern China, rice is consumed as the main staple food and a major source of nutrients for the poor who lack access to diverse foods. Recently, concern has been raised about possible contamination of the crop by heavy metals.

Many industrialized processes give rise to the contamination by heavy metals, such as cadmium (Cd), mercury (Hg) and lead (Pb) in soil, water and air. The contaminants can be accumulated and transferred in rice. Fu et al. [3] found the Pb with the mean level of 0.69 mg/kg in polished rice in a typical electronic waste recycling area from the southeast China. Zhao et al. [4] reported the Cd with a maximum value of 0.467 mg/kg in rice. Huang et al. [5] also observed the high Pb level of 0.957 mg/kg in rice. All these studies indicated the possible contamination of rice by heavy metals.

Undoubtedly, the high exposure of these metals had the confirmative negative effects to human health [6], [7]. Cd is toxic to the kidney and has a long biological half-life in human. Pb has shown to be associated with damage of central nervous system, leading to decrements of intelligence quotients in children. As regards with the toxicity of Hg, especially methylmercury, the central nervous system is the main target organ, particularly during foetal development. To the general population, the dietary intake is the main exposure pathway. It is therefore reasonable to hypothesize that rice as the staple food containing heavy metals have the potential health risk to consumers.

Zhejiang province, partly belong to Yangtze River Delta of China, is a rapidly developing region with a high population density, where heavy metal is one of the most important environmental issues [8]. Previous study has revealed the heavy metal pollution in the HJH (Hangzhou–Jiaxing–Huzhou) water-network plain in Yangtze River Delta [9], [10]. However, to our knowledge, few studies on the level of heavy metals in rice and exposure assessment in Zhejiang were reported.

The main aims of this study were to analyze heavy metals in rice from Zhejiang province and evaluate the health risk with respect to daily consumption of rice for general adults and children. The results of our study may provide some insight into heavy metal accumulation in rice and serve as a basis for comparison to other regions both in China and worldwide.

## Materials and Methods

### Rice Consumption data

The rice consumption data used in this report was extracted from the Food Consumption Survey conducted in Zhejiang province, China in 2008 by the Zhejiang Food and Drug Administration [11]. The representative sample of participants included 9798 people, who were questioned twice about their last 24-h consumption. The selection of interviewed people and the moment of the interview were chosen in order to obtain a representative consumption profile of the population over 1 year. The estimated rice intakes of adult (18 years old or over) and children (7–18 years old) were 342.90 g/day per person and 258.43 g/day per person.

### Sampling and sample preparation

**Figure 1:**
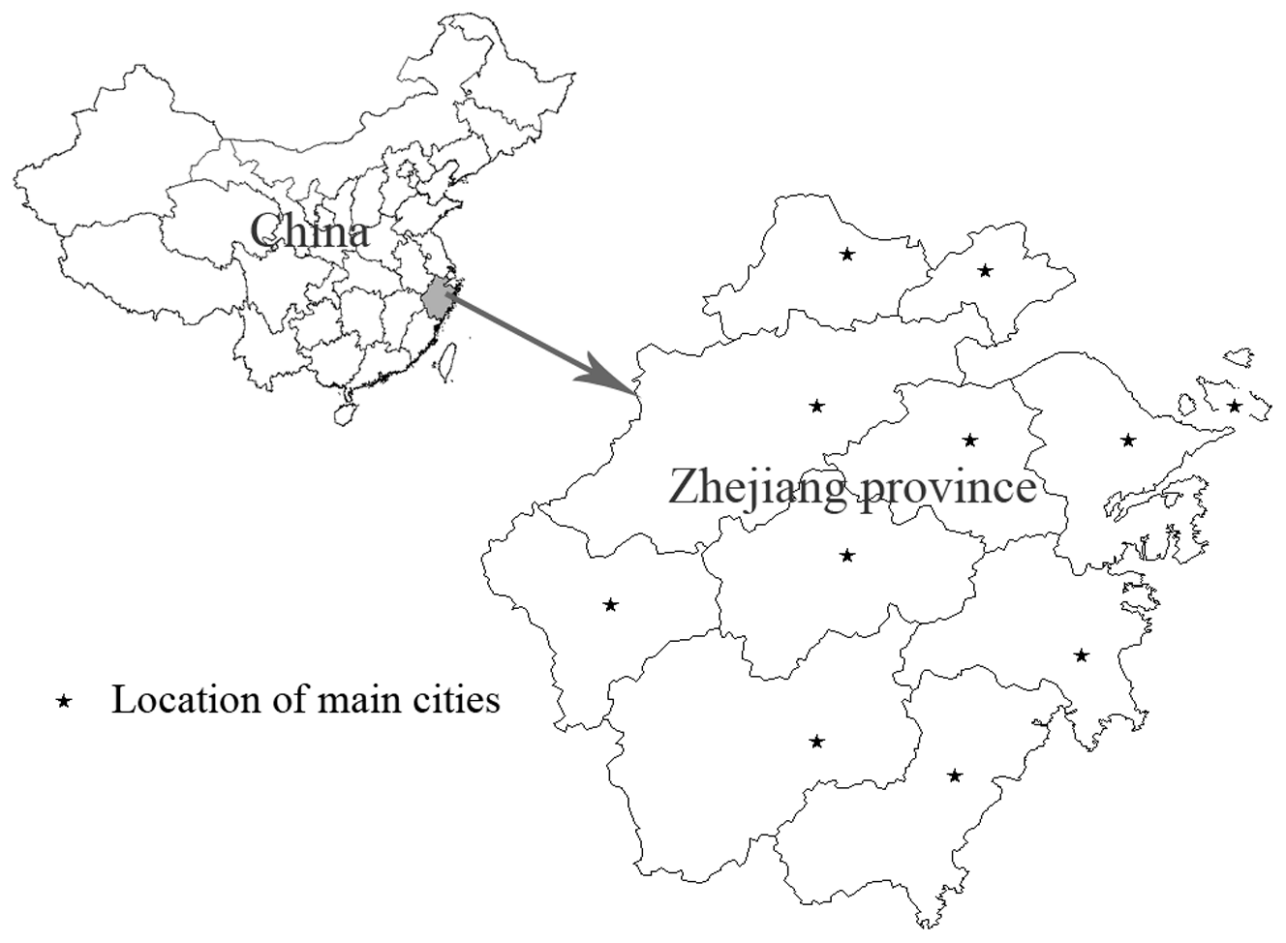
Simple map of the sampling areas of Zhejiang province, China.

Total 248 rice samples (polished) were collected from local markets in May to October, 2012. The sampling place of Zhejiang was shown in Figure 1. Total 11 sites marked with asterisks in Zhejiang province were included. The samples were collected and kept in plastic bags and frozen for further analysis.

### Chemical analysis

For the determination of heavy metals, samples were digested as follows: a 1–2 g sample was poured to a 100 mL round-bottom flask. Then, 10 mL of concentrated nitric acid was added to the sample and heated at 120°C. 1 mL of hydrogen peroxide was periodically added to the solution until the digestion step was complete, i.e., a clear solution was reached. Usually, 2–2.5 mL of hydrogen peroxide was sufficient. After that we transferred the solution into a 50 mL volumetric flask and filled with distilled water. The solution was prepared for analysis of heavy metals.

The levels of As, Cd, Pb, and Hg were determined according to the methods described by Husain et al. [12] and Fu et al. [13]. A Thermo SOLAAR model iCE3000 atomic absorption spectrometry (AAS) with a graphite furnace was used for the analysis of Cd and Pb in the prepared samples. The As and Hg concentration was determined by hydride generation-atomic fluorescence spectrometry (HG-AFS 9230, Jitian Co., Beijing, China).

### Analysis of CRMs

The accuracy of the analytical procedures was verified by analysis of appropriate certificated reference materials (CRMs) using the same digestion and analytical methods. Two CRMs (Table 1) were purchased from National Research Center for Certified Reference Materials, China (NRCCRM). Quantitative results (within 10% of the certified value) were obtained for each metal in each CRM. Recoveries were ranged between 91–105%. Limits of Detection (LODs) were defined as 3 times the standard deviation of 10 runs of blank measurements. LODs of As, Cd, Hg and Pb were 0.005, 0.001, 0.005 and 0.005 mg/kg respectively.

**Table 1:** Determination of certified materials of rice.

	GBW10044			GBW08502		
	Certifiedmg/kg	Measuredmg/kg	Recovery(%)	Certifiedmg/kg	Measuredmg/kg	Recovery(%)
As	0.12±0.03	0.11±0.05	91	0.051 ± 0.003	0.048±0.005	94
Cd	0.018± 0.002	0.019±0.004	105	0.020 ± 0.002	0.019±0.005	95
Hg	2.2 ± 0.5	2.1±0. 8	95	—	—	—
Pb	0.09±0.03	0.09±0.04	100	0.75 ± 0.05	0.74±0.04	98

### Exposure estimates

The data used for exposure estimates were according to the recommendation of the report Reliable Evaluation of Low-Level Contaminations of Food issued by WHO after the 2^nd^ GEMS/Food-EURO Workshop 1995 [14]. Thus, a value of ^1^/_2_ LOD was assigned to all results below the LOD, where the proportion of <LOD results is not >60%.

Exposure from rice was obtained by combining its consumption data and the heavy metal concentrations of the specific item and then dividing by body weight. The average body weight in this study was considered as 55.9 kg for adult (18 years old or over) and 32.7 kg for children (7–18 years old) [15]. The mean and 97.5th percentile of the daily exposure levels were used to represent the dietary exposure for average and high consumers, respectively [16]. The health risk index was calculated by dividing daily intake of heavy metals by their safe limits [17]. An index more than 1 was considered as not safe for human health [18].

## Results and Discussion

### Heavy metals in rice

Of all the rice samples we measured, the highest concentrations of As, Cd, Hg and Pb were found to be 0.189, 0.112, 0.088 and 0.220 mg/kg respectively. The mean levels, P97.5 and the range were listed in Table 2. The comparison of these metals in rice with some previous studies was showed in Table 3. The extent of rice contamination can be evaluated by comparing with the maximum allowable concentrations (MAC) recommended by Chinese legislation [19], [20].

**Table 2:** The concentration of heavy metals in rice from Zhejiang province (mg/kg).

Elements	n	Mean±SD^a^	P95^a^	range	MAC^b^	No. of > MAC
As	248	0.080±0.051	0.166	<LOD–0.246	0.7	0
Cd	248	0.037±0.015	0.105	<LOD–0.112	0.2	0
Hg	224	0.005±0.003	0.060	<LOD–0.088	0.02	15
Pb	248	0.060±0.034	0.206	0.005–0.220	0.2	9

a Target analytes with concentrations lower than LOD were treated as one-half of LOD when calculating the mean values; SD, standard deviation.

b Maximum allowable concentrations of contaminants in foods [19], [20].

**Table 3:** Comparison of the levels of heavy metals in rice and exposure estimates to some previous studies.

	area	N	Mean levelmg/kg	Exposureµg/kg bw/day	References
As	China	712	0.119(LOD-0.490)	—	Qian et al. [28]
	China (Changshu)	155	0.199(LOD-0.587)	1.4 (adults) 1.2 (Children)	Huang et al. [5]
	China (Taizhou)	13	0.155(0.095–0.308)	0.8	Fu et al. [3]
	Taiwan	204	0.080	—	Lin et al. [23]
	Turkey	25	0.098 (0.0204–0.1708)	—	Gunduz et al. [24]
	U.S.	112	0.20	—	Zavala et al. [26]
	Spanish	24	0.21	—	Torres-Escribano et al. [25]
Cd	China	712	0.050(LOD-0.740)	—	Qian et al. [28]
	China (Jiangsu)	23	0.014(0.005–0.032)	—	Cao et al. [31]
	China (Changshu)	155	0.019(LOD-0.201)	0.1(adults) 0.1(Children)	Huang et al. [5]
	China (Taizhou)	13	0.224(0.012–0.661)	0.7	Fu et al. [3]
	China	269	0.081(–0.340)	—	Chen et al.[30]
	Iran	67	0.062(0.038-0.122)	—	Shakerian et al.[29]
	Turkey	25	0.031(0.0084–0.0775)	—	Gunduz et al.[24]
Hg	China	712	0.006(LOD-0.031)	—	Qian et al. [28]
	China (Jiangsu)	23	0.006(0.001–0.013)	—	Cao et al. [31]
	China (Changshu)	155	0.014(LOD-0.060)	0.1(adults) 0.1(Children)	Huang et al. [5]
	China (Taizhou)	13	0.022(0.016–0.068)	0.1	Fu et al. [3]
	Brazil	44	0.3–13.4	0.2	Batista et al. [32]
Pb	China	712	0.062(LOD-0.400)	—	Qian et al. [28]
	China (Jiangsu)	23	0.054(0.0076–0.12)	—	Cao et al.[31]
	China (Changshu)	155	0.171(LOD-0.957)	1.2(adults) 1.0(children)	Huang et al. [5]
	China (Taizhou)	13	2.042(0.256–2.602)	3.7	Fu et al. [3]
	China	269	0.114(–1.136)	—	Chen et al.[30]
	BrazilIran	4467	0.4 to 14.50.068(0.040–0.128)	0.4	Shakerian et al.[29]

#### Arsenic (As)

It was reported that arsenic can be easily accumulated by all types of cereals, largely because of the high bioavailability of arsenic under reduced soil conditions [21]. Rice is much more efficient at assimilating arsenic into its grain than other staple cereal crops [22]. In this survey, total arsenic concentration varied over a range of <LOD to 0.206 mg/kg with a mean of 0.080 mg/kg. The data from all samples were lower than the current MAC of 0.7 mg/kg. This result was similar with the data observed in Taiwan (0.80 mg/kg) and Turkey (0.98 mg/kg) [23], [24]. A high mean level of 0.199 mg/kg was found in Changshu, China by Huang et al. [5]. Even higher level of 0.2 mg/kg in rice was reported both in U.S. and Spanish [25], [26]. The concentration of arsenic in rice mainly depends on the condition of the paddy soil. Paddy soils can become elevated in arsenic from a number of anthropogenic diffuse and point sources of contamination.

As we known, inorganic species (iAs) were thought to be the most toxic in As species. It is reported that iAs in Asian and European rice was the dominate species (about 30 to 100% iAs) [26], [27]. According to MAC (0.2 mg/kg) for iAs in China, there could be 8 rice samples (3%) with the iAs levels exceeding the MAC in this study.

#### Cadmium (Cd)

The concentration of Cd was found at the mean of 0.037 mg/kg (<LOD–0.112 mg/kg). All samples contained lower level than the MAC of 0.2 mg/kg. The result was similar with the report by Qian et al. [28], who observed the mean level of 0.050 mg/kg (*n = *712) in China. A survey of Iran rice found a mean level of 0.062 mg/kg (*n* = 67) [29]. In Turkey, 0.031 mg/kg (*n = *25) were found in rice [24]. In recent years, several studies reported that the mean levels of Cd were no more than 0.1 mg/kg in common area of China [5], [30], [31]. However, high levels of Cd in rice ranging from 0.21 to 2.4 mg/kg were revealed in some contaminated sites of China [3], [32].

#### Mercury (Hg)

The Hg level was observed with a mean of 0.005 mg/kg (<LOD–0.088 mg/kg). According to the current MAC of 0.02 mg/kg, 93.4% of total samples were acceptable on Hg contamination level. Our results were lower than those reported in other area of China which were ranged from 0.006 mg/kg to 0.014 mg/kg [5], [28], [31]. A high mean level of 0.022 mg/kg (*n = *13) in the polluted area of China, was reported by Huang et al. [5]. Also, the high Hg level ranged from 0.3 to 13.4 mg/kg (*n = *44) in rice was reported in Brazil [32].

Mercury is a widespread pollutant and a threat to human health. Methylmercury (MeHg) is known as a strong toxicant. The nervous system is the primary target organ for MeHg poisoning and the brain of developing fetus is more sensitive than that of the adults [33]. Previous study has highlighted that rice can accumulate relatively high levels of MeHg (0.174 mg/kg) in Hg mining areas in Guizhou, Southwestern China [34]. Although the level of Hg found in this study was low, concerns still need to be made in terms of the potential pollution.

#### Lead (Pb)

The mean level of Pb was 0.060 mg/kg ranged from 0.005 to 0.220 mg/kg. According to the current MAC of 0.02 mg/kg, 96.4% of total samples were acceptable on Pb contamination level. As shown in Table 3, the reports on Pb levels in rice from China varied with a great extent. Similar levels with our data were reported by Qian et al. [28] and Cao et al. [31]. Chen et al. [30] and Huang et al. [5] found the Pb with the mean level of 0.114 mg/kg and 0.171 mg/kg respectively. In polluted area of China, the mean Pb level was as high as 2.042 mg/kg (*n = *13) [3]. The variance might be caused by the different sampling sites and pollution status.

### Spatial distribution of heavy metals

**Figure 2:**
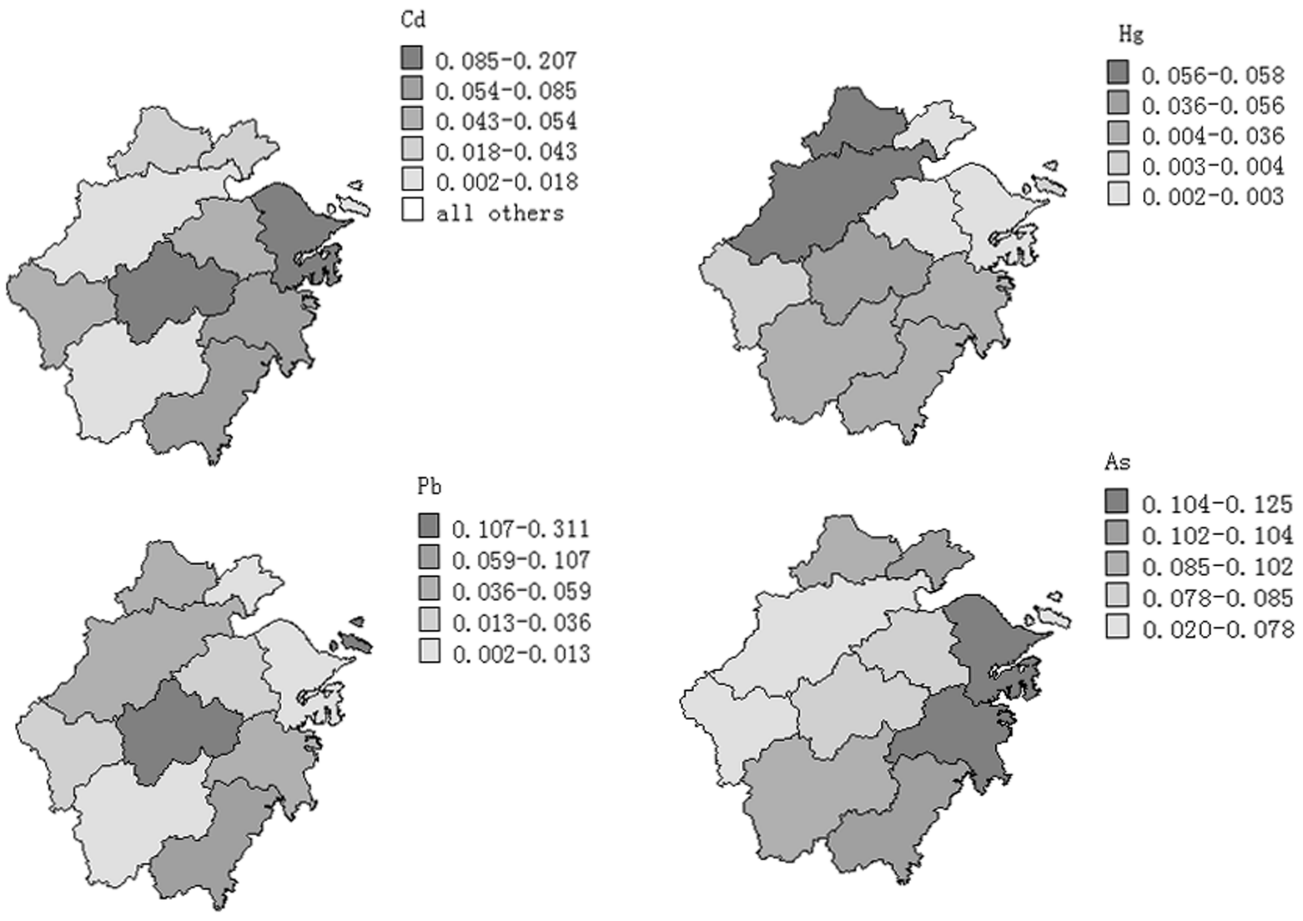
The spatial distribution of the As, Cd, Hg and Pb in rice from Zhejiang province, China.

The spatial distribution of these metals in Zhejiang province was shown in Figure 2. The highest Pb level was found in middle area, and the highest As level was in the east zones. Both middle and east area showed the high Cd level in rice. No regular distribution of As, Cd, Hg and Pb in rice was found in areas of Zhejiang. Another study also reported that the spatial patterns of heavy metals in rice were irregular in their geographical distribution [5].

### Estimated daily intake (EDI) of heavy metals

Although other pathways of human exposure to heavy metals were referred, such as air and water, rice consumption was considered the major one. The EDIs of adults and children were showed in Table 4. The 97.5th percentile (P97.5) level was used to represent the high exposed consumers of the distribution. The mean intakes of As, Cd, Hg and Pb through rice are estimated to be 0.49, 0.23, 0.03 and 0.37 µg/kg bw/day for adults, and 0.34, 0.29, 0.04 and 0.47 µg/kg bw/day for children. The P97.5 daily intakes of As, Cd, Hg and Pb were 1.02, 0.64, 0.37 and 1.26 µg/kg bw/day for adults, and 0.63, 0.83, 0.47 and 1.63 µg/kg bw/day for children. Comparing with the recommended safe value (showed in Table 4), the P97.5 daily intakes of Hg for adults and Pb for children exceeded the safe limit. It indicates that the long-term large consumption of rice will result in the high exposure of Hg and Pb in Zhejiang.

**Table 4:** Estimated exposure to As, Cd, Pb and Hg for the general population in rice from Zhejiang province and the percentage of the safe value.

	Safe valueµg/kg bw/day	Intake of Adultsµg/kg bw/day		%(Mean/P97.5)	Intake of Childrenµg/kg bw/day		%(Mean/P97.5)
		Mean	P97.5		Mean	P97.5	
As	3.0^a^	0.49	1.02	0.16/0.34	0.34	0.63	0.21/0.44
Cd	0.8^b^	0.23	0.64	0.28/0.77	0.29	0.83	0.35/1.00
Hg	0.14 (adults)^c^0.57 (children)^c^	0.03	0.37	0.21/2.64	0.04	0.47	0.07/0.82
Pb	1.5^d^	0.37	1.26	0.25/0.84	0.47	1.63	0.31/1.09

a The provisional tolerable weekly intake (PTWI) of 21 µg/kg bw (equivalent to 3 µg/kg bw/day) according to JECFA [35].

b PTMI 0.025 mg/kg bw on a monthly basis according to JECFA [36].

_c_ For adults (1 µg/kg bw per week) and for children (4 µg/kg bw per week) according to JECFA [35].

d Based on cardio-vascular effects according to EFSA [37].

### Health risk to adults and children

The health risk index (HI) described by the percentage of the safe value was used for the risk assessment. As shown in Table 4, for the mean exposure, the data of HI were all less than 1, which indicated that there was no potential health risk to general people. However, the HI based on the P97.5 estimate had the value not less than 1 (Table 4), such as the Cd and Pb for children. The P97.5 HI of Hg for adults was high and up to 2.64. Concerns might be paid for the high exposure of Hg by the rice consumption for adults.

It is not surprising that the P97.5 intakes of heavy metals were high. As shown in Table 3, previous studies revealed that rice contained the high level of heavy metals in some special areas [3], [34]. However, the low levels of heavy metals in rice were also reported in China [28], [31]. The diverse data in China were probably caused by the two factors: (1) irregular designed industry zones where the contaminants can not be all controlled efficiently; (2) non-intensive rice cultivation with small scale which can lead to the discrepancy of quality in rice.

## Conclusion

The mean levels of As, Cd, Hg and Pb in rice from Zhejiang, China were all below their MAC of China. Also, the mean daily exposures were estimated to be safe on contrast with the values of the tolerable intake set by the JECFA or EFSA. We concluded that the consumption of rice from Zhejiang had no obvious health risk to general children and adults for these heavy metals. However, the P97.5 estimates of Cd and Pb for children, and Hg for adults have exceeded the respective safe limits, which indicated that these people, especially those living in contaminated locations, may experience some adverse health effects.

Accordingly, the regular monitoring of heavy metals in rice is recommended in this area. The factors leading to the different levels of heavy metals will be investigated in our future studies.
